# Biomimetic Catalysts Based on Au@TiO_2_-MoS_2_-CeO_2_ Composites for the Production of Hydrogen by Water Splitting

**DOI:** 10.3390/ijms24010363

**Published:** 2022-12-26

**Authors:** Kenneth Fontánez, Diego García, Dayna Ortiz, Paola Sampayo, Luis Hernández, María Cotto, José Ducongé, Francisco Díaz, Carmen Morant, Florian Petrescu, Abniel Machín, Francisco Márquez

**Affiliations:** 1 Department of Chemistry, University of Puerto Rico, Rio Piedras Campus, San Juan 00925, Puerto Rico; 2 Department of Biochemistry, School of Medicine, University of Puerto Rico, Medical Sciences Campus, San Juan 00936, Puerto Rico; 3 Nanomaterials Research Group, Department of Natural Sciences and Technology, Division of Natural Sciences, Technology and Environment, Universidad Ana G. Méndez-Gurabo Campus, Gurabo 00778, Puerto Rico; 4 Department of Applied Physics, Autonomous University of Madrid, Instituto de Ciencia de Materiales Nicolás Cabrera, 28049 Madrid, Spain; 5 Department of Natural Sciences and Technology, Division of Natural Sciences, Technology and Environment, Universidad Ana G. Méndez-Cupey Campus, San Juan 00926, Puerto Rico

**Keywords:** hydrogen production, TiO_2_, gold nanoparticles, MoS_2_, CeO_2_, water splitting

## Abstract

The photocatalytic hydrogen evolution reaction (HER) by water splitting has been studied, using catalysts based on crystalline TiO_2_ nanowires (TiO_2_NWs), which were synthesized by a hydrothermal procedure. This nanomaterial was subsequently modified by incorporating different loadings (1%, 3% and 5%) of gold nanoparticles (AuNPs) on the surface, previously exfoliated MoS_2_ nanosheets, and CeO_2_ nanoparticles (CeO_2_NPs). These nanomaterials, as well as the different synthesized catalysts, were characterized by electron microscopy (HR-SEM and HR-TEM), XPS, XRD, Raman, Reflectance and BET surface area. HER studies were performed in aqueous solution, under irradiation at different wavelengths (UV-visible), which were selected through the appropriate use of optical filters. The results obtained show that there is a synergistic effect between the different nanomaterials of the catalysts. The specific area of the catalyst, and especially the increased loading of MoS_2_ and CeO_2_NPs in the catalyst substantially improved the H_2_ production, with values of ca. 1114 μm/hg for the catalyst that had the best efficiency. Recyclability studies showed only a decrease in activity of approx. 7% after 15 cycles of use, possibly due to partial leaching of gold nanoparticles during catalyst use cycles. The results obtained in this research are certainly relevant and open many possibilities regarding the potential use and scaling of these heterostructures in the photocatalytic production of H_2_ from water.

## 1. Introduction

There is a global concern about the present and future consequences of climate change. One of the main focuses in the last decade has been cutting or reducing the dependence of fossil fuels to meet our energy requirements [1,2]. Hydrogen, as an energy vector [3], is a promising candidate because it can be obtained from renewable sources like water, its combustion products are mainly water or water vapor, is less toxic than gasoline or any other usual fuel, among others [4,5].

Photosynthesis is considered the best and most efficient model that allows the conversion of solar energy for the generation of clean fuel. In nature, photosynthesis operates by supplying electrons to the active center of photosystem-II. This process is carried out through four consecutive steps of proton-coupled electron transfer, generating, as a final result of the process, products derived from reduced carbon that are the basis of life and biological activity. Considering the inspiration of this natural process, continuous efforts have been made for decades to implement and assemble some of these photosynthetic mechanisms in order to use solar energy to generate oxygen and hydrogen by splitting water [6,7,8].

One of the methods to produce hydrogen via water splitting is by photocatalysis [9,10,11]. Usually semiconductors including titanium oxide (TiO_2_), zinc oxide (ZnO), iron (III) oxide (Fe_2_O_3_), zinc sulfide (ZnS), zirconium oxide (ZrO_2_), cadmium sulfide (CdS), among others, are selected as photocatalysts due to their narrow bandgap and electronic structure [12,13,14,15]. TiO_2_ is one of the most studied and used catalysts in photocatalysis for the reduction of water and degradation of organic pollutants [16], although it presents some disadvantages: (1) Recombination of photo-generated electron hole pairs [17]; (2) Fast backward reaction [18]; and (3) Inability to use visible light. The band gap of TiO_2_ is 3.2 eV for anatase, 3.0 eV for rutile, and 3.4 eV for brookite, and with this band gap energy, only ultraviolet light (UV) can be used for hydrogen production [19]. To work with these limitations, multiple chemical modifications have been developed and implemented over the years. 

One of them is the incorporation of noble metals, such as silver (Ag), gold (Au) and platinum (Pt), on the surface of titanium oxide due to the ability of the noble metal nanoparticles in reducing the fast recombination of the photogenerated charge carriers, enabling the use of visible light [20]. By reducing the photogenerated charge carriers, the UV activity is increased due to the electron transfer from the CB of TiO_2_ to the noble metal nanoparticles [21]. The photoactivity in the visible range of the electromagnetic spectrum can be explained due to the surface plasmon resonance effect and charge separation by the transfer of photoexcited electrons from the metal nanoparticles to the CB of TiO_2_ [22]. Obtaining heterostructures by coupling two or more materials with different properties makes it possible to improve the photocatalytic activity of the system [23,24]. Among these heterostructures, it is worth highlighting TiO_2_-ZnO, TiO_2_-WO_3_, TiO_2_-CdS, TiO_2_-SO_2_, among others, which have shown considerable improvements compared to the materials used separately [23,24]. A semiconductor that has gained popularity in recent years has been cerium (IV) oxide, CeO_2_. As with TiO_2_, CeO_2_ has a high bandgap energy (from 2.6 eV to 3.4 eV, depending on the synthesis process and the material obtained), and high thermal stability. CeO_2_ can be synthesized with different morphologies, it can be doped with metal or non-metal ions, it can be combined with other materials to form more efficient heterostructures, and it can be used for a wide variety of catalytic processes [25]. The incorporation of metal dichalcogenides, such as molybdenum disulfide (MoS_2_), has also been explored to replace the use of noble metal co-catalysts due to its high abundance, low cost, good stability, and high catalytic activity [26]. However, the use of MoS_2_ could also reduce the charge transfer rate and, therefore, the efficiency in some catalytic hydrogen production processes [27].

As shown above, there is no perfect catalyst to produce hydrogen by water splitting. Therefore, the objective of this research has been to explore the capabilities of different materials to produce hydrogen under visible and ultraviolet light and to combine these to obtain catalytically active heterostructures. To achieve this, different cocatalysts were incorporated onto TiO_2_. The fifteen photocatalysts synthesized are made up of gold nanoparticles (Au NPs; 1%, 3%, 5% by weight), cerium (IV) oxide nanoparticles (CeO_2_ NPs; 1%, 3%, 5% by weight), and molybdenum disulfide (MoS_2_; 1%, 3%, 5% by weight), and have been fully characterized and evaluated in the photocatalytic reaction of hydrogen production by water splitting.

## 2. Results and Discussion

### 2.1. Characterization of Catalysts

Fifteen catalysts were synthesized, based on Au nanoparticles deposited on TiO_2_ nanowires (TiO_2_NWs), MoS_2_, and CeO_2_ nanoparticles (CeO_2_NPs). The proportion of Au nanoparticles, as well as MoS_2_ and CeO_2_NPs, were conveniently varied, considering TiO_2_NWs as the base component. These catalysts were used for the hydrogen evolution reaction (HER) from the photocatalytic decomposition of water, and the most efficient catalyst (3%Au@TiO_2_NWs-5%MoS_2_-5%CeO_2_NPs) was fully characterized by different techniques.

Table S1 shows the BET surface area of the different components and of the synthesized catalysts. As can be seen, TiO_2_NWs shows a high surface area of 236 m^2^/g that increases with the incorporation of Au nanoparticles on the surface, going from 242 m^2^/g (1%Au@TiO_2_NWs) to 263 m^2^/g (3%Au@TiO_2_NWs), and to 275 m^2^/g (5%Au@TiO_2_NWs). This effect of increasing the area by incorporating nanoparticles has been previously described [28]. The other two components of the synthesized catalysts (MoS_2_ and CeO_2_NPs) also show high areas that justify the high area values observed in the catalysts, which range between 248 and 396 m^2^/g. As can be seen in Table S1, in general, the surface area increases with the addition of Au, although this trend is not so clear with the increase of the other two components.

**Figure 1:**
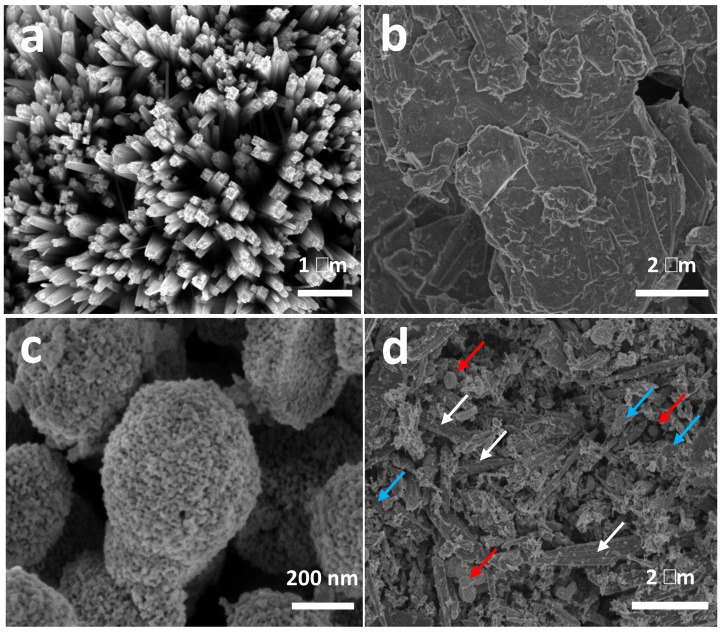
HR-SEM micrographs of TiO_2_NWs (**a**), MoS_2_ nanosheets (**b**), CeO_2_NPs (**c**) and the 3%Au@TiO_2_NWs-5%MoS_2_-5%CeO_2_NPs catalyst (**d**). The arrows in (**d**) indicate the different components of the catalyst: TiO_2_NWs (white), MoS_2_ (blue), and CeO_2_NPs (red).

**Figure 2:**
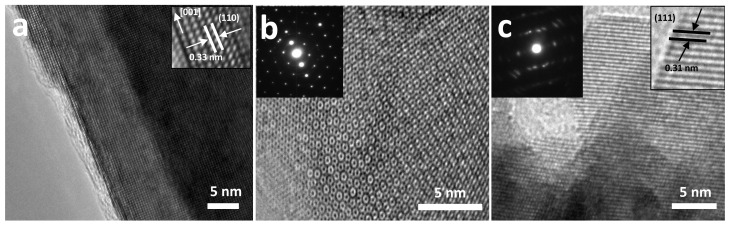
HR-TEM micrographs of the different components of the catalysts: TiO_2_NWs and inset at atomic resolution showing the direction of growth and the lattice fringes (**a**); MoS_2_ single layer and inset corresponding to the selected area electron diffraction, SAED (**b**); and CeO_2_NPs and insets corresponding to SAED and micrograph at higher magnification showing the lattice fringes (**c**).

The catalyst precursors were characterized by electron microscopy. Figure 1a shows the HR-SEM image of TiO_2_NWs, characterized by being formed by square-section wires, with diameters ranging from ca. 200 to 300 nm and lengths of up to 10 μm. MoS_2_, previously delaminated by prolonged ultrasound treatment, shows a layered structure with variable lengths from 1 to 2 μm (see Figure 1b). The effect of exfoliation of MoS_2_, by high power ultrasound, can be seen in the HR-SEM micrograph of Figure S1, taken at low magnification. Figure S1 shows a MoS_2_ particle in an intermediate stage of delamination, and before the layers have dispersed. CeO_2_NPs are characterized by presenting spherical aggregates of more than 400 nm which, in turn, are formed by very homogeneous nanoparticles with sizes ranging from 4 to 6 nm (Figure 1c). Figure 1d shows the HR-SEM image of the catalyst that showed the highest efficiency (3%Au@TiO_2_NWs-5%MoS_2_-5%CeO_2_NPs). As can be seen in Figure 1d, the different components of the catalyst show a good dispersion. The components of the studied catalysts were characterized by HR-TEM. Figure 2a shows the atomic resolution image of TiO_2_NWs. The material is highly crystalline, showing the distinct lattice fringes with an interplanar spacing of 0.33 nm, indexed to (110) crystal plane which corresponds to the rutile phase [29]. On the other hand, the growth of TiO_2_NWs takes place along (001) direction determined by HR-TEM image, which is consistent with XRD results that will be discussed later. MoS_2_ shows a high level of exfoliation (Figure 2b), which allows us to observe the detail of the atomic structure of a monolayer. As can be seen in the inset of Figure 2b, corresponding to the selected area electron diffraction (SAED), the material is highly crystalline. Apparently, and although it is still to be confirmed, the HR-TEM analyses seem to indicate the presence of structural defects generated by the appearance of vacancies in the two-dimensional structure of the material. These defects could be due to the intense exfoliation process produced by high intensity ultrasound, and could be related to the high activity of the catalysts. Figure 2c shows the HR-TEM image of CeO_2_NPs. As seen in the SAED, the material is crystalline. Part of the image has been further magnified to show detail of the lattice fringes, with an interplanar spacing of 0.31 nm, indexed to (111) crystal plane corresponding to the characteristic face-centered cubic fluorite-type structure [30].

**Figure 3:**
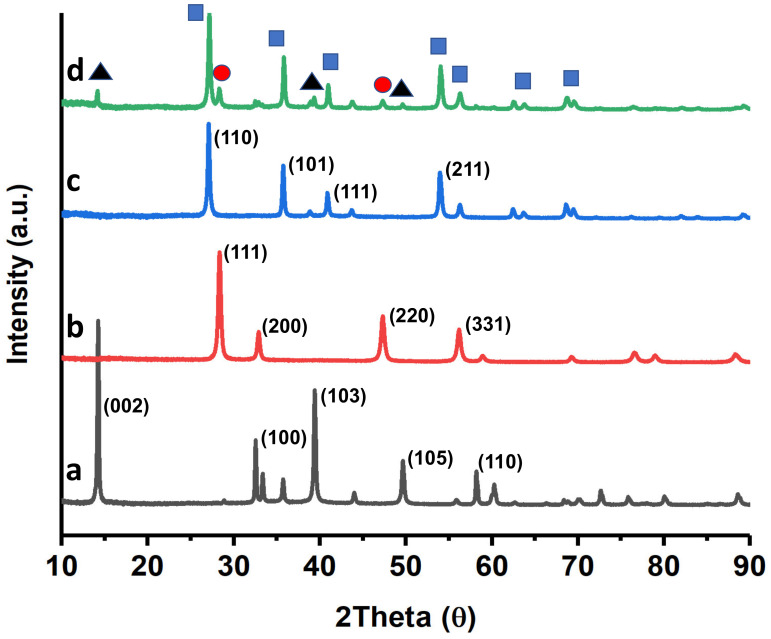
XRD patterns of MoS_2_ (**a**); CeO_2_NPs (**b**); TiO_2_NWs (**c**); and 3%Au@TiO_2_NWs-5%MoS_2_-5%CeO_2_NPs (**d**). The most intense peaks in the catalyst have been associated with the different components (black triangles: MoS_2_, red circles: CeO_2_NPs, blue squares: TiO_2_NWs).

Figure 3 shows the results obtained by X-ray diffraction (XRD) for the most efficient catalyst (3%Au@TiO_2_NWs-5%MoS_2_-5%CeO_2_NPs), along with that of MoS_2_, CeO_2_NPs and TiO_2_NWs for comparison purposes. As shown in Figure 3a, well-defined diffraction peaks are observed at ca. 14°, 32°, 39°, 49°, and 59° that have been ascribed to (002), (100), (103), (105), and (110) planes of 2H-type MoS_2_ hexagonal phase (JCPDS # 75–1539), respectively [31,32,33]. Figure 3b shows the diffraction pattern of CeO_2_NPs. The most intense peaks are observed at ca. 28°, 33°, 47° and 56°, and correspond to (111), (200), (220) and (311) crystal planes, respectively [34,35,36]. These peaks are characteristic of CeO_2_ with face-centered cubic fluorite-type structure. Figure 3c shows the XRD pattern of TiO_2_NWs. The diffraction peaks at ca. 27°, 36°, 41°, y 54° were ascribed to (110), (101), (111), y (211) TiO_2_ crystalline planes in rutile phase (JCPDS 75-1750) [37,38]. The XRD of the most efficient catalyst is shown in Figure 3d. As can be seen there, the main peaks of all three components are present. However, the presence of Au, which should be shown as a very low intensity peak at ca. 38° [39], corresponding to Au (111), is not observed in the catalyst, possibly due to the high dispersion of the metal. 

**Figure 4:**
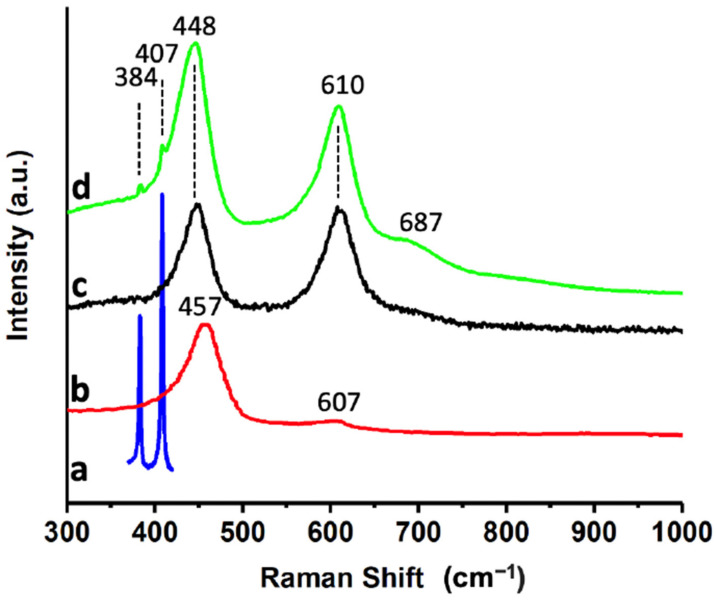
Raman spectra of MoS_2_ (**a**); CeO_2_NPs (**b**); TiO_2_NWs (**c**); and 3%Au@TiO_2_NWs-5%MoS_2_-5%CeO_2_NPs (**d**).

The different materials, as well as the most efficient catalyst, were characterized by Raman spectroscopy (Figure 4). MoS_2_ shows two very characteristic bands at 383 cm^−1^ and 407 cm^−1^ (Figure 4a), which have been assigned to the E^1^_2g_ and A_1g_ modes, respectively [40]. The position of these bands has been correlated with the number of layers of the material, so the results suggest that the exfoliation process was very efficient, generating MoS_2_ flakes with few layers [41,42]. CeO_2_NPs (Figure 4b) shows an intense band at ca. 457 cm^−1^, and a much less pronounced one at 607 cm^−1^ that have been assigned to a cubic fluorite structure, as already evidenced from the XRD results. The main band at 457 cm^−1^ corresponds to a triply degenerate F_2g_ mode of symmetric stretching vibrations of oxygen ions around Ce^4+^ ions in octahedral CeO_8_ [43]. The asymmetry of the band at 457 cm^−1^ has been associated with structural defects due to the presence of oxygen vacancies in the oxide [44,45], which could also be correlated with the reactivity of the material. Figure 4c shows the Raman spectrum of TiO_2_NWs, whose bands at ca. 448 cm^−1^ and 610 cm^−1^ have been assigned to the vibration modes E_g_ and A_1g_ of TiO_2_ in the rutile phase, as already evidenced by XRD. The Raman spectrum of the most efficient catalyst (Figure 4d) shows two pronounced bands at 448 cm^−1^ and 610 cm^−1^, and a shoulder at ca. 687 cm^−1^. These three bands come from rutile, which is the major component of the catalyst. Additionally, two small peaks are observed at ca. 384 cm^−1^ and 407 cm^−1^ assigned to MoS_2_. Due to the position of the CeO_2_NPs bands, the signal of this material is masked under the strong contribution of rutile.

**Figure 5:**
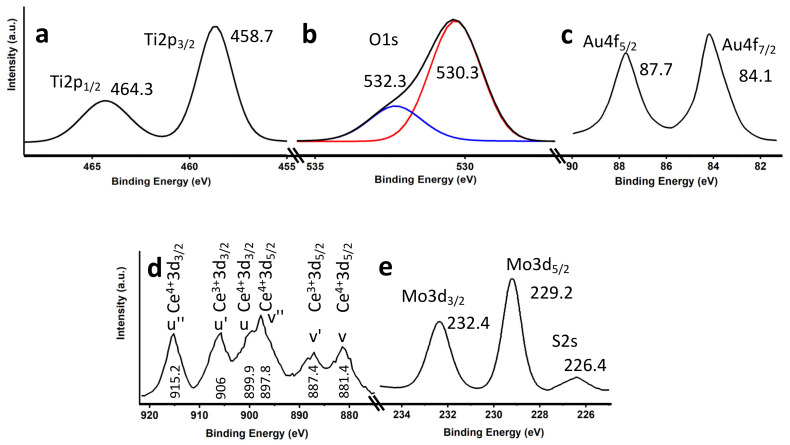
XPS core level spectra for Ti2p (**a**); O1s (**b**); Au4f (**c**); Ce3d (**d**); and Mo3d/S2s (**e**).

The most efficient catalyst (3%Au@TiO_2_NWs-5%MoS_2_-5%CeO_2_NPs) was also characterized by X-ray photoelectron spectroscopy (XPS). Ti2p (Figure 5a) shows two components at 464.3 eV and 458.7 eV that were ascribed to the Ti2p_1/2_ and Ti2p_3/2_ transitions, respectively [3,9]. These transitions are quite symmetrical, so any additional contribution was ruled out. Figure 5b shows the transition corresponding to O1s. As can be seen, the transition is clearly asymmetric and has been deconvoluted into two components at ca. 530.3 eV and 532.3 eV. The most intense peak (530.3 eV) has been assigned to oxygen in the TiO_2_ lattice [3,46], which also masks the possible contribution of oxygen in the CeO_2_ lattice, while the component observed at 532.3 eV has been assigned to oxygen vacancies in CeO_2_ [47] (as suggested by the asymmetry of the main peak of CeO_2_NPs in Raman spectroscopy), or to non-lattice oxygen [46]. Figure 5c shows the Au4f transition, with peaks at 84.1 eV and 87.7 eV and a characteristic spin-orbit splitting of ca. 3.6 eV, which have been clearly assigned to the presence of metallic Au [48]. The transition corresponding to Ce3d is shown in Figure 5d. This transition, which is very complex due to a state hybridization process, evidences two distinguishable series of peaks corresponding to the Ce^4+^ and Ce^3+^ species. The different peaks were labeled u, u′, u″, v, v′ and v″ to represent the different electronic states of Ce^4+^ and Ce^3+^ [47,49]. The presence of Ce^3+^ ions gives rise to a charge imbalance, responsible for oxygen vacancies and the presence of defects and unsaturated chemical bonds in the nanomaterial. These defects in CeO_2_NPs support the results previously shown by Raman and XPS.

Figure 5d shows the Mo3d and S2s transitions. The Mo3d shows two peaks at 232.4 eV and 229.2 eV, which have been attributed to the Mo3d_3/2_ and Mo3d_5/2_ doublet, respectively, characteristic of the Mo^4+^ state in MoS_2_ [48,50]. The observed peak at ca. 226.4 eV was assigned to S2s [48], typical of MoS_2_. The slight asymmetry of the Mo3d peaks could point to a possible mixture of oxidation states, which could also be correlated with the potential presence of defects in the material lattice and reactivity.

**Figure 6:**
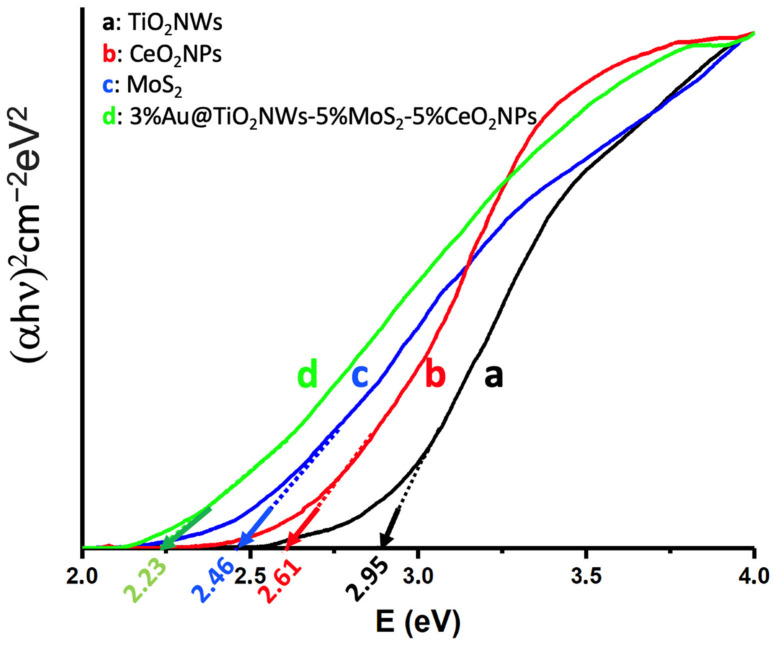
Tauc plots of (αhn)^2^ versus energy (eV), and determination of the bandgap energy of TiO_2_NWs (**a**); CeO_2_NPs (**b**); MoS_2_ (**c**); and 3%Au@TiO_2_NWs-5%MoS_2_-5%CeO_2_NPs (**d**).

The efficiency of radiation absorption by the catalysts is a critical factor for their activity, so the different catalysts and nanomaterials used were analyzed using Tauc diagrams [51]. As shown in Figure 6, TiO_2_NWs showed a bandgap in the border region between UV and visible (2.95 eV), slightly different from the expected value for TiO_2_ in rutile phase (3.05 eV) [52]. CeO_2_NPs and MoS_2_ show bandgaps at 2.61 eV and 2.46 eV, respectively, clearly in the visible region. The most efficient catalyst, formed as a heterostructure of these components in addition to the presence of Au nanoparticles, shows a bandgap at 2.23 eV (ca. 555 nm), which clearly justifies the activity of the 3%Au@TiO_2_NWs-5%MoS_2_-5%CeO_2_NPs heterostructure under irradiation with visible light, as will be described in the section corresponding to catalytic results.

### 2.2. Photocatalytic Hydrogen Production

Before proceeding to evaluate the activity of the synthesized catalysts, several preliminary studies were carried out to establish the optimal reaction conditions. To do this, we started from the heterostructure with the highest proportion of each of the components (5%Au@TiO_2_NWs-5%MoS_2_-5%CeO_2_NPs). Initially, a study of the optimum pH was carried out (see Figure S2a), and it was established that the most suitable was pH = 7. Another of the preliminary studies that was carried out allowed establishing the optimum amount of catalyst in the reaction medium. As can be seen in Figure S2b, there is a clear correlation between the amount of catalyst and the HER efficiency, so a loading of 50 mg of catalyst (for a total reaction volume of 100 mL) was established as the optimal amount.

**Figure 7:**
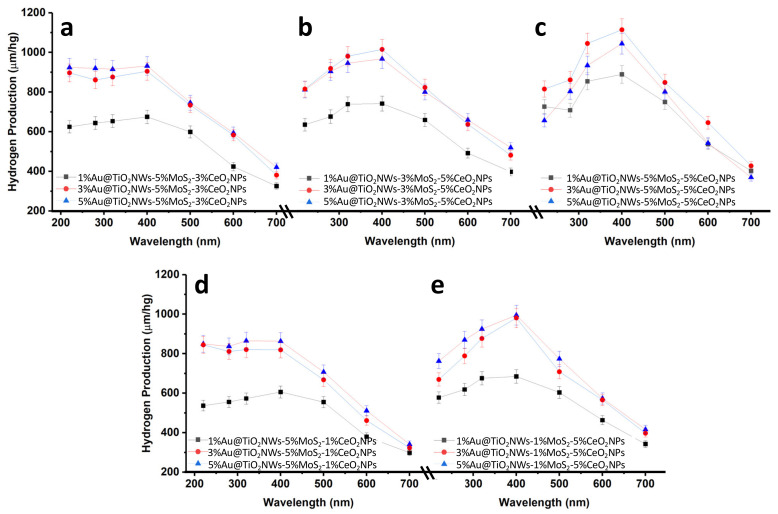
H_2_ production profiles of the synthesized catalysts under irradiation at different wavelengths (the legends corresponding to the catalysts are shown in each of the figures). The estimated error bars for each of the values obtained are also shown.

The photocatalytic efficiency of the catalysts for the evolution of H_2_ was evaluated in the presence of Na_2_SO_3_ (0.02 M) and Na_2_S (0.4 M) as sacrificial reagents. In all cases, it was found that there is no evolution of H_2_ without either photocatalyst or irradiation (see Figure S2c). Figure 7 shows the results of the photocatalytic hydrogen production from the fifteen synthesized catalysts. In all cases, the activity under irradiation with different wavelengths (220, 280, 320, 400, 500, 600 and 700 nm) was evaluated. As can be seen, the maximum hydrogen production is observed under irradiation at 400 nm, this wavelength being the one corresponding to the bandgap of TiO_2_NWs (see Table S2). At more energetic wavelengths the behavior varies from one system to another. Figure 7a,d, corresponding to the heterostructures Au@TiO_2_NWs-5%MoS_2_-3%CeO_2_NPs and Au@TiO_2_NWs-5%MoS_2_-1%CeO_2_NPs, respectively, show a similar trend under irradiation at 220, 280 and 320 nm, with stable values of hydrogen production in catalysts with 3% and 5% Au. Catalysts with lower Au loading (1%) show much lower activities. In all the catalysts a decrease in activity is observed under irradiation in the visible range (λ > 400 nm), although surprisingly hydrogen production is observed even at very low energy wavelengths (i.e., 700 nm). This behavior clearly points to a synergy between the different nanomaterials, and specifically to the gold load, which, as observed, has a significant effect on the levels of hydrogen production. The highest catalytic activity is observed with 3%Au@TiO_2_NWs-5%MoS_2_-5%CeO_2_NPs, showing a hydrogen production of 1114 μm/hg under irradiation at 400 nm. In contrast, the catalyst with the lowest efficiency was the Au@TiO_2_NWs-5%MoS_2_-1%CeO_2_NPs heterostructure, and specifically the one with 1%Au, with only a hydrogen production of 606 μm/hg (Figure 7d). This behavior is evidence of the relevance of CeO_2_NPs in the heterostructure. As previously discussed in Section 2.1, CeO_2_NPs present oxygen vacancies and defects that may be responsible for the reactivity of the material. These defects, as already described [47], might originate from the effective separation of photogenerated electron–hole pairs within the composite endorsing the charge transfer efficiency. The effect of MoS_2_ on the catalyst activity is indisputable, although it is not as relevant as for CeO_2_NPs. When comparing the catalysts Au@TiO_2_NWs-1%MoS_2_-5%CeO_2_NPs (Figure 7e), Au@TiO_2_NWs-3%MoS_2_-5%CeO_2_NPs (Figure 7b) and Au@TiO_2_NWs-5%MoS_2_-5%CeO_2_NPs (Figure 7c) it is observed that by increasing the amount of MoS_2_ the production of H_2_ also increases substantially. This effect is more notable when going from 3% to 5% of MoS_2_ in the heterostructure. MoS_2_ has high surface area values, as shown in Table S1. The increase in MoS_2_ loading produces an increase in the specific area of the heterostructure, together with the improvement in the conductivity properties of the catalyst [53], which could justify the effect of this nanomaterial on the reactivity observed in Figure 7. On the other hand, the presence of Au, and especially the generation of surface plasmons generated by the Au nanoparticles on the surface of the heterostructure [54], represents an adjuvant factor on the photocatalytic HER.

The recyclability of the most efficient catalyst (3%Au@TiO_2_NWs-5%MoS_2_-5%CeO_2_NPs) was also evaluated. For this, 15 consecutive reactions were carried out with the same catalyst (Figure S3) using a larger reaction volume (200 mL). After each reaction, the catalyst was recovered by centrifugation (3000 rpm, 15 min), washed with deionized water, and dried overnight in an oven at 50 °C. The recovered catalyst was used again in the next reaction, using the same experimental conditions, and keeping the reaction temperature constant at 20 °C. After 15 cycles of use, the results obtained (Figure S3) showed an efficiency of ca. 93% of the initial, which represents a loss of activity of around 7%. After cycle 15, the catalyst showed similar morphological characteristics although, according to EDX measurements, the gold loading was slightly reduced to approximately 2.4%, which could indicate a gold leaching effect during the use cycles, that would justify the loss of 7% of activity. This result is remarkable considering that the catalysts are heterostructures formed by four components whose synergistic behavior remains almost unchanged in each cycle of use.

Some results of recent studies on hydrogen production by water splitting using heterostructured catalysts are shown in Table S3 [55,56,57,58,59,60,61,62]. As can be seen there, the amount of hydrogen reported in our research is one of the highest, although a direct comparison with other research is not possible because the experimental conditions, reaction time, and even nanomaterials are not the same. Nonetheless, our hydrogen production results are certainly promising, and could potentially even be applicable to larger-scale processes.

### 2.3. Mechanism of the Photocatalytic Hydrogen Production

Figure S4 shows the effect of incorporating hole scavengers (methanol and EDTA-Na_2_) on HER. Figure S4a to Figure S4e show the most efficient systems in the absence of scavengers and in the presence of each of them. As can be seen, the addition of methanol (5 mL) to the reaction medium clearly increases the production of hydrogen at all wavelengths. The effect of the addition of EDTA-Na_2_ (0.1 M) produces an even greater effect, with pronounced increases in all the systems used and practically under any irradiation energy. The incorporation of larger amounts of methanol or EDTA-Na_2_ did not produce significant changes, therefore, at least for these hole scavengers and for the reaction conditions used, the maximum possible H_2_ production was reached. These results clearly suggest that electron-hole recombination occurs in the absence of scavengers, despite the effect of electron channeling towards the Au nanoparticles and MoS_2_ nanosheets from the catalysts and the use of electron donors (Na_2_SO_3_, 0.02 M and Na_2_S, 0.4 M) in the reaction mixture. 

**Figure 8:**
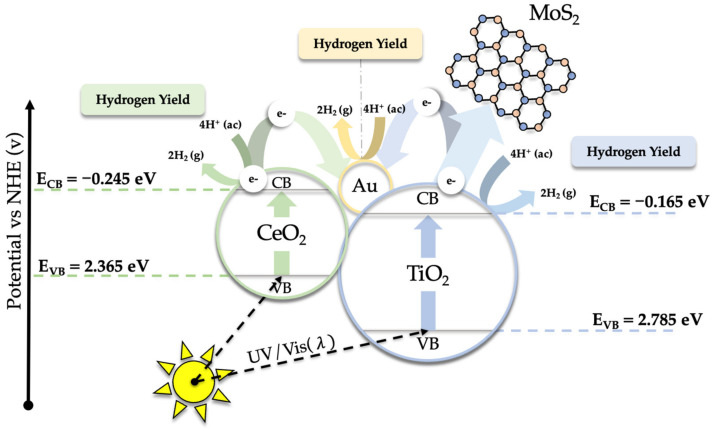
Mechanism proposed for the hydrogen production using Au@TiO_2_NWs-MoS_2_-CeO_2_NPs catalysts under UV-visible irradiation.

Considering these results, in addition to the determination of bandgaps (see Figure 6), a tentative mechanism for HER has been proposed (Figure 8). To do this, Mulliken’s classical theory of electronegativity has been used [63,64], which makes it possible to establish the position of the edge of the band of the different nanomaterials that form the heterostructure and, based on it, to establish the direction of migration of the photogenerated charge carriers in the catalyst (see Equations (1) and (2)).

E_CB_ = χ − E_C_ − 0.5E_g_ (1)

E_VB_ = E_CB_ + E_g_ (2)

where E_CB_ and E_VB_ are the edge potentials of the valence band and the conduction band, respectively, χ is the absolute electronegativity, E_C_ is the energy of free electrons on the hydrogen scale (4.50 eV) [65,66], and E_g_ is the experimentally determined bandgap (see Figure 6). The X values for TiO_2_ (rutile) and CeO_2_ are 5.81 and 5.56 eV, respectively [67,68]. The E_CB_ and E_VB_ edge positions for TiO_2_NWs determined from Equations (1) and (2) are −0.165 and 2.785 eV, respectively, while for CeO_2_NPs the calculated values were −0.245 and 2.365 eV (see Figure 8).

As previously shown [58], the presence of CeO_2_NPs and MoS_2_ significantly broadens the range of light absorption, which increases the density of photogenerated electrons and the production of H_2_. Under irradiation, the valence band electrons of both TiO_2_NWs and CeO_2_NPs are photoexcited to their corresponding conduction bands [9]. These electrons can reduce the water, generating H_2_, or be transferred to the MoS_2_ or Au nanoparticles. Both MoS_2_ and Au act as sinks that channel electrons, prevent electron-hole recombination, and facilitate subsequent reactivity [3,11,69]. The holes that were created in the valence band of the heterostructure, as previously shown, undergo partial recombination with the photogenerated electrons, although this recombination is inhibited by adding hole scavengers (methanol or EDTA-Na_2_) to the reaction mixture. As previously described, the presence of Ce^3+^ and Ce^4+^ species, identified by XPS (see Figure 5d), may play a relevant role in prolonging the lifetime of photoinduced charge carriers [58]. Ce^4+^ species can trap the electrons helping to avoid electron-hole recombination. On the other hand, Ce^3+^/oxygen vacancies can provide abundant H_2_O adsorption sites, which decreases the H_2_O adsorption energy, increasing the efficiency of the water splitting reaction. 

## 3. Materials and Methods

### 3.1. Reagents and Materials

All reagents were used as received without further purification. All solutions were prepared with deionized water (Milli-Q water, Burlington, MA, USA, 18.2 MΩcm^−1^ at 25 °C). TiCl_4_ (99.9%) was provided by Fisher Scientific, Cayey, Puerto Rico. HAuCl_4_·3H_2_O (ACS Reagent, St. Louis, MO, USA, 49.0 + % Au basis), MoS_2_ (Nanopowder, St. Louis, MO, USA, 90 nm diameter, 99% trace metals basis), Ce(NO_3_)_3_·6H_2_O (99.99%), Ethanol (200 proof, anhydrous, ≥99.5%), EDTA disodium salt dihydrate (OmniPur, St. Louis, MO, USA), and NaBH_4_ (99.99% trace metals basis) were provided by Sigma Aldrich (Darmstadt, Germany). Methanol anhydrous for UHPLC-MS LiChrosolv (99.9%) was provided by Supelco (Bellefonte, PA, USA). Silicon p-type boron doped substrates (Si <100>), were provided by El-CAT (Ridgefield Park, NJ, USA). UHP N_2_ (5.0), used for the photocatalytic reaction, was provided by Praxair, Gurabo, Puerto Rico.

### 3.2. Synthesis of Nanomaterials

The synthesis of titanium oxide nanowires (TiO_2_NWs) has been previously described [70]. In a typical synthesis, a mixture of water and HCl (37% solution) (1:1, *v/v*) was prepared. Subsequently, TiCl_4_ (3 mL) was added dropwise to 100 mL of the solution and allowed to mix for 30 min until the presence of suspended particles was not observed. The solution was then transferred to Teflon-lined autoclaves, and silicon substrates (Si <100>) with the polished surface facing the inside of the Teflon container, were incorporated into the solution. The autoclaves were then sealed and transferred to an oven. The autoclaves were treated at 180 °C for 24 h. Once the treatment time had elapsed, the autoclaves were left to cool for at least 12 h. As a result, the growth of a white deposit on the surface of the Si substrates was observed. The material obtained was washed with abundant deionized water, dried in an oven at 60 °C and stored in vials that were sealed until later use.

The deposition of gold nanoparticles (AuNPs) was carried out by dispersing 1 g of the support (TiO_2_NWs), whose synthesis was previously described, in 100 mL of H_2_O and the mixture was sonicated for 30 min. Next, the required amount of gold precursor (HAuCl_4_·3H_2_O) was added to the reaction mixture and stirred for 1 h. Finally, the process continued with the reduction of gold by adding, dropwise, a NaBH_4_ solution (10 mg in 10 mL of H_2_O) under constant stirring. Once the 10 mL of NaBH_4_ had been added, the resulting solution was kept under stirring for 1 h. The reaction product was separated by centrifugation (3000 rpm, 15 min), washed 4 times with deionized water, and dried overnight at 60 °C. The different Au@TiO_2_NWs compounds were synthesized with 1%, 3% and 5% AuNPs on the surface and these materials were later used for the incorporation of the rest of the catalyst components.

CeO_2_ nanoparticles (CeO_2_NPs) were obtained through a coprecipitation process. For this, two solutions were prepared: (i) 250 mL of a solution of Ce(NO_3_)_3_·6H_2_O (0.02 M) and (ii) 250 mL of a solution of K_2_CO_3_ (0.03 M). Both solutions were introduced dropwise into an Erlenmeyer flask containing 50 mL of water. During this process, the mixture was kept under constant stirring. As a result, a precipitate of Ce_2_(CO_3_)_3_ was obtained, which was separated from the solution by centrifugation. The resulting solid was washed four times with deionized water and dried at 70 °C for 3 h. Next, the dry material was calcined in a muffle at 600 °C for 3 h, using an open crucible.

The commercial MoS_2_ was subjected to an exfoliation process before being used. For this, 4 g of MoS_2_ were mixed with 200 mL of deionized water. The resulting dispersion was sonicated using a Cole-Palmer Tip Sonicator (Cole-Parmer 750-Watt Ultrasonic Processor) for 6 h in pulsed mode (40% amplitude, pulse on 5 s, pulse off 10 s). Subsequently, the solution was kept static for sedimentation for 3 h. Next, the supernatant was extracted from the mixture and centrifuged for 30 min at 3000 rpm to remove the non-delaminated MoS_2_. Next, the supernatant can be dried by evaporation at 50 °C to be used later, or manipulated directly as a suspension. The concentration of the suspension can be determined by measuring the absorbance at 672 nm, using the Beer-Lambert law, and considering ε as 3400 mL mg^−1^ m^−1^.

The materials, whose synthesis has been described above, were used for the following stages of preparation of the catalysts. Thus, 300 mg of Au@TiO_2_NWs were dispersed in a solution containing 20 mL of ethanol and 20 mL of deionized water, and the mixture was vigorously stirred for 1 h. Subsequently, cerium oxide nanoparticles (CeO_2_NPs) were added, and the suspension was stirred for 2 h. The product was then separated from solution by centrifugation (3000 rpm, 15 min), washed 4 times with deionized water, and dried overnight at 60 °C. The incorporation of MoS_2_ was carried out in a final synthesis step, and by a procedure similar to that previously described for CeO_2_NPs. Once the synthesis process was finished, the product was recovered by centrifugation (3000 rpm, 15 min), washed four times with deionized water, dried overnight at 60 °C, and stored and sealed at room temperature until later use. The 15 synthesized catalysts, based on Au@TiO_2_NWs-MoS_2_-CeO_2_NPs, were identified indicating the percentage of gold incorporated on the surface and the percentages of MoS_2_ and CeO_2_NPs in each case.

### 3.3. Characterization of the Catalysts

The surface morphology of the catalysts was evaluated using a FEI Verios 460 L High Resolution Scanning Electron Microscope (HR-SEM, Thermo Fisher Scientific, Hillsboro, OR, USA), equipped with a Quantax EDS Analyzer, and by High Resolution Transmission Electron Microscopy (HR-TEM), using a JEOL JEM 3000F (300 kV) microscope. XPS measurements were carried out using an ESCALAB 220i-XL spectrometer, using non-monochromatic Mg Kα (1253.6 eV) radiation from a twin anode, operating at 20 mA and 12 kV in the constant analyzer energy mode, with a PE of 50 eV. The crystallinity of the catalysts was studied by X-ray diffraction, using a Bruker D8-Advance diffractometer that operates at 40 kV and 40 mA in the range of 20–80°, using a Bragg-Brentano configuration, and at a scan speed of 1° min^−1^. The catalysts were also characterized by Raman spectroscopy, using a DXR Thermo Raman Microscope, which uses a 532 nm laser source at 5 mW power and a 25 µm pinhole aperture with a 5 cm^−1^ nominal resolution. Bandgap measurements of the different materials were carried out using a Perkin Elmer Lambda 365 UV-Vis spectrophotometer (Perkin Elmer, Waltham, MA, USA), equipped with an integrating sphere. The bandgap value was obtained from the graph of the Kubelka-Munk function versus the absorbed light energy [51]. Brunauer Emmett Teller (BET) specific area measurements were carried out using a Micromeritics ASAP 2020 system, according to N_2_ adsorption isotherms at 77 K.

### 3.4. Photocatalytic Hydrogen Production

The experimental setup for the characterization of the catalysts for the hydrogen evolution reaction (HER) by photocatalytic water splitting consisted of mixing 50 mg of the desired catalyst with 100 mL of deionized water in a 200 mL quartz reactor. Next, sacrificial electron donor solutions (Na_2_SO_3_, 0.02 M; Na_2_S, 0.4 M) were added. In order to test the effect of adding additional hole scavengers to the reaction mixture, methanol (5 mL) and EDTA-Na_2_ (0.1 M) were used. The reaction mixture was kept at 20 °C for 1 h before the start of the reaction, to guarantee temperature stability, and was purged with nitrogen (N_2_, 5.0) during the pre-reaction process. Next, the reaction mixture was irradiated using a solar simulator, whose irradiation power in the absence of filters is 120 mW.cm^−2^). To study the influence of irradiation energy on the water splitting reaction, different cut-off filters at 220, 280, 320, 400, 500, 600, and 700 nm were used, and the reaction was followed for two hours. The hydrogen produced was quantified using a gas chromatograph coupled to a thermal conductivity detector (GC-TCD, Perkin-Elmer Clarus 600).

## 4. Conclusions

A total of 15 catalysts with different amounts of Au, MoS_2_, and CeO_2_ (1%, 3%, and 5% by weight) incorporated onto TiO_2_NWs were synthesized, and their photocatalytic activity was evaluated by the production of hydrogen via water splitting using visible and ultraviolet light. The highest hydrogen production was 1114 μm/hg, and was obtained with the 3%Au@TiO_2_NWs-5%MoS_2_-5%CeO_2_NPs composite. The combination of the different materials caused a synergistic effect, increasing the catalytic activity and allowing the use of wavelengths ranging from 220 to the visible range, with remarkable efficiency even under irradiation at wavelengths as low in energy as 700 nm. The recyclability test showed an efficiency loss of ca. 7% after 15 cycles, suggesting a stable and suitable catalyst for the photocatalytic production of hydrogen by water splitting. 

The results obtained in this research are certainly the starting point for further developments that allow us to delve into the mechanisms that control the HER. In this sense, the continuation of this research, already in progress, will analyze three factors that, in our opinion, are of great relevance for the catalytic systems studied: (i) the effect of increasing the loading of MoS_2_ and CeO_2_NPs in the heterostructure, plus beyond the 5% considered in the present investigation; (ii) analysis of how the use of cerium oxides in which the Ce^3+^/Ce^4+^ ratio can be modulated influences HER; and (iii) characterization of possible leaching during catalyst use and regeneration cycles.

## Data Availability

The data is contained in the article and is available from the corresponding authors on reasonable request.

## Supplementary Materials

The following supporting information can be downloaded at: https://www.mdpi.com/article/10.3390/ijms24010363/s1.
