# Supplementary material for: Biomimetic Catalysts Based on Au@TiO2-MoS2-CeO2 Composites for the Production of Hydrogen by Water Splitting
Source: Int J Mol Sci. 2022 Dec 26;24(1):363. doi: 10.3390/ijms24010363 (PMC9820641; doi:10.3390/ijms24010363)
Supplement: Supplementary file 1 [file ijms-24-00363-s001.zip › ijms-2092584-supplementary.pdf]

Supplementary Information

Biomimetic Catalysts Based on $Au@TiO_2$-MoS_2-CeO_2 Composites for the Production of Hydrogen by Water Splitting

Kenneth Fontánez [1], Diego García [2], Dayna Ortiz [3], Paola Sampayo [3], Luis Hernández [3], María Cotto [3], José Ducongé [3], Francisco Díaz [3], Carmen Morant [4], Florian Petrescu [3], Abniel Machín [5,*], and Francisco Márquez [3,*]

[1] Department of Chemistry, University of Puerto Rico, Rio Piedras Campus, San Juan, 00925PR, United States
[2] Department of Biochemistry, School of Medicine, University of Puerto Rico, Medical Sciences Campus, San Juan, 00936PR, United States
[3] Nanomaterials Research Group, Department of Natural Sciences and Technology, Division of Natural Sciences, Technology and Environment, Universidad Ana G. Méndez-Gurabo Campus, 00778PR, United States
[4] Department of Applied Physics, Autonomous University of Madrid, and Instituto de Ciencia de Materiales Nicolás Cabrera, 28049, Madrid, Spain
[5] Department of Natural Sciences and Technology, Division of Natural Sciences, Technology and Environment, Universidad Ana G. Méndez-Cupey Campus, 00926PR, United States
* Correspondence: fmarquez@uagm.edu (F.M.), machina1@uagm.edu (A.M.)

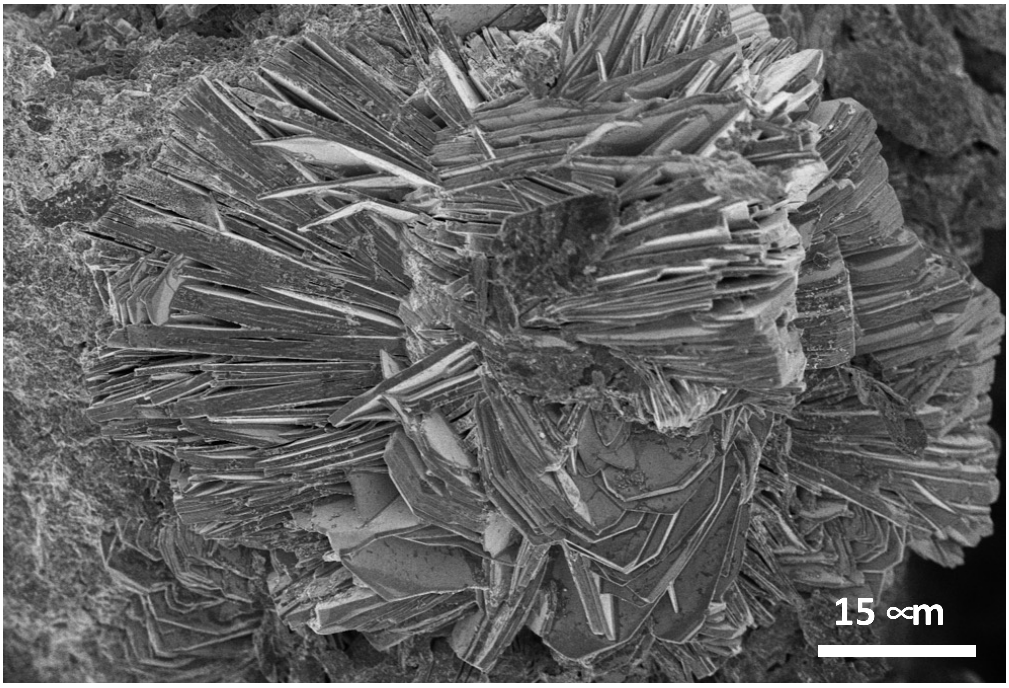

Figure S1. HR-SEM micrograph of a MoS_2 particle in an intermediate stage of exfoliation, and before being dispersed.

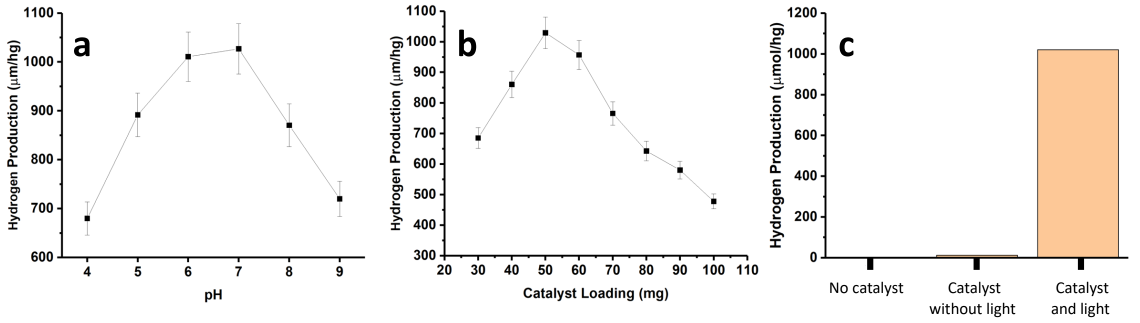

Figure S2. Evaluation of the initial concentration of the catalyst (5%Au@TiO_2NWs-5%MoS_2-5%CeO_2NPs) (a) and of the pH (b) on the H_2 production; and control experiments for 3%Au@TiO_2NWs-5%MoS_2-5%CeO_2NPs (c).

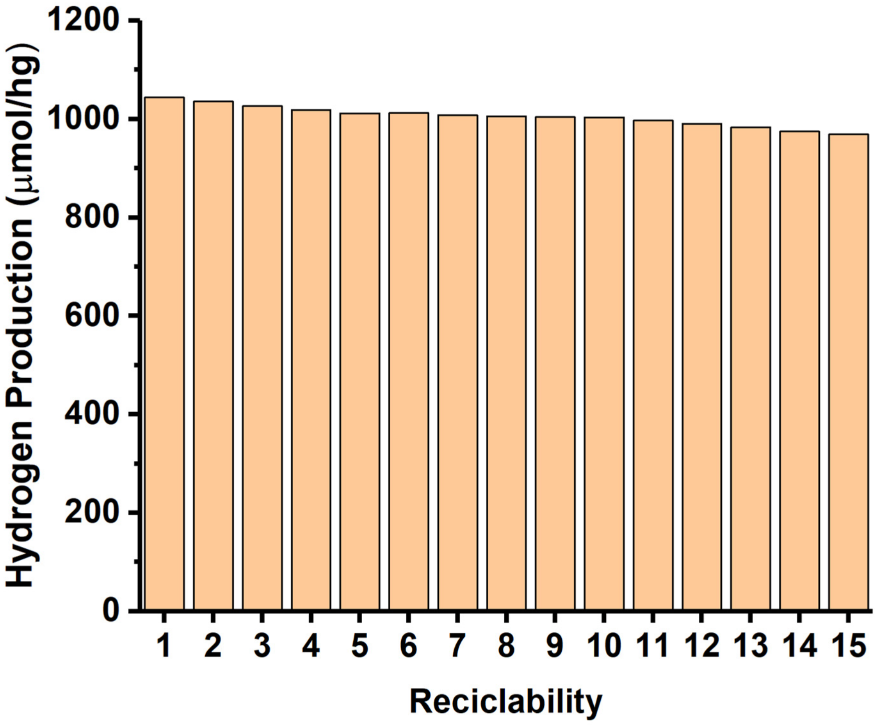

Figure S3. Recyclability of 5%Au@TiO2NWs-5%MoS2-5%CeO2NPs after 15 consecutive catalytic HER cycles under UV-visible radiation. The results obtained are affected by 5% error.

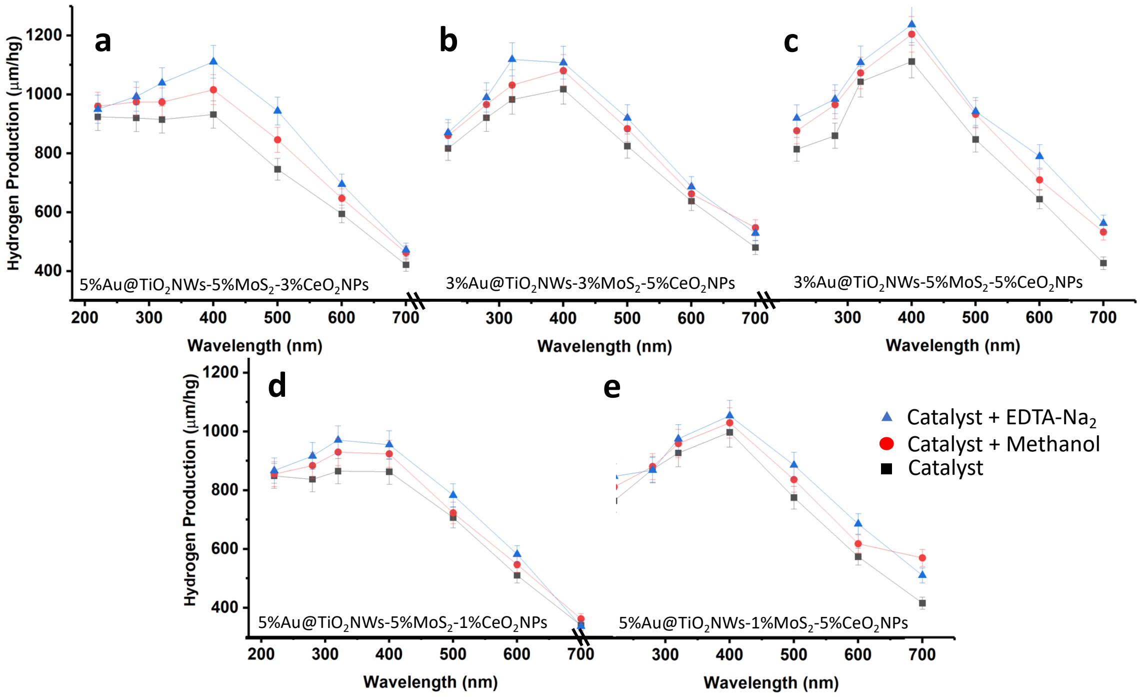

Figure S4. Photocatalytic activity of the most representative types of catalysts in the presence of hole scavengers (methanol and EDTA-Na2) under UV-vis radiation. Black symbols show the results obtained with the catalyst alone. The symbols in red and blue color show the results obtained with the catalyst in the presence of methanol and EDTA-NA2 respectively.

Table S1. BET surface area of the as-synthesized materials.

Material	BET area (m^2/g)
TiO_2NWs	236
1%Au@TiO_2NWs	242
3%Au@TiO_2NWs	263
5%Au@TiO_2NWs	275
MoS_2	183
CeO_2NPs	241
1%Au@TiO_2NWs-1%MoS_2-5%CeO_2NPs	311
3%Au@TiO_2NWs-1%MoS_2-5%CeO_2NPs	327
5%Au@TiO_2NWs-1%MoS_2-5%CeO_2NPs	252
1%Au@TiO_2NWs-3%MoS_2-5%CeO_2NPs	301
3%Au@TiO_2NWs-3%MoS_2-5%CeO_2NPs	322
5%Au@TiO_2NWs-3%MoS_2-5%CeO_2NPs	329
1%Au@TiO_2NWs-5%MoS_2-1%CeO_2NPs	289
3%Au@TiO_2NWs-5%MoS_2-1%CeO_2NPs	295
5%Au@TiO_2NWs-5%MoS_2-1%CeO_2NPs	307
1%Au@TiO_2NWs-5%MoS_2-3%CeO_2NPs	248
3%Au@TiO_2NWs-5%MoS_2-3%CeO_2NPs	261
5%Au@TiO_2NWs-5%MoS_2-3%CeO_2NPs	272
1%Au@TiO_2NWs-5%MoS_2-5%CeO_2NPs	332
3%Au@TiO_2NWs-5%MoS_2-5%CeO_2NPs	347
5%Au@TiO_2NWs-5%MoS_2-5%CeO_2NPs	396

Table S2. Maximum H_2 production (µmol/hg) by the synthesized catalysts under irradiation at different wavelengths. All values obtained are affected by 5% error.

Catalyst	Irradiation Wavelength (nm)						
	**220**	**280**	**320**	**400**	**500**	**600**	**700**
1%Au@TiO_2NWs-1%MoS_2-5%CeO_2NPs	577	618	675	684	603	463	342
3%Au@TiO_2NWs-1%MoS_2-5%CeO_2NPs	669	788	876	980	708	565	397
5%Au@TiO_2NWs-1%MoS_2-5%CeO_2NPs	762	869	924	993	773	571	416
1%Au@TiO_2NWs-3%MoS_2-5%CeO_2NPs	637	678	741	744	661	493	398
3%Au@TiO_2NWs-3%MoS_2-5%CeO_2NPs	818	922	984	1019	826	639	480
5%Au@TiO_2NWs-3%MoS_2-5%CeO_2NPs	814	907	949	971	803	661	521
1%Au@TiO_2NWs-5%MoS_2-1%CeO_2NPs	537	556	573	606	555	380	298
3%Au@TiO_2NWs-5%MoS_2-1%CeO_2NPs	845	811	821	819	668	462	323
5%Au@TiO_2NWs-5%MoS_2-1%CeO_2NPs	849	837	865	863	707	511	341
1%Au@TiO_2NWs-5%MoS_2-3%CeO_2NPs	625	644	653	675	599	424	325
3%Au@TiO_2NWs-5%MoS_2-3%CeO_2NPs	897	861	876	905	733	583	381
5%Au@TiO_2NWs-5%MoS_2-3%CeO_2NPs	924	920	915	932	741	594	420
1%Au@TiO_2NWs-5%MoS_2-5%CeO_2NPs	726	708	854	889	749	536	402
3%Au@TiO_2NWs-5%MoS_2-5%CeO_2NPs	815	861	1045	1114	848	645	427
5%Au@TiO_2NWs-5%MoS_2-5%CeO_2NPs	656	803	934	1044	801	542	369

Table S3: Recent research on photocatalytic hydrogen production using heterostructured catalysts.

Catalyst	H_2 Production	Reference
3%Au@ZnO-5%MoS_2-5%CeO_2	1114 μmol/hg	This work
ZnCdS@Co-MoS_2	551.48 μmol/h	[1]
Au@CeO_2	1.6-8.6 μmol/hmg	[2]
Pd@TiO_2	979 μmol/hg	[3]
CeO_2@MoS_2/g-C_3N_4	65 μmol/h	[4]
B-doped CN_4/ZnO	357 μmol/hg	[5]
$SrTiO_3$@TiO_2@Fe_2O_3	716 μmol/cm^3	[6]
3DOM SnS_2-ZnS/ZrO_2	928.1 μmol	[7]
Au-CuO_2	153 μmol/h g	[8]

References

1. Yonggang, L.; Yingzhen, Z.; Zengxing, L.; Shen, X.; Jianying, H.; Kim, H.N.; Yuekun, L. Molybdenum Sulfide Cocatalyst Activation upon Photodeposition of Cobalt for Improved Photocatalytic Hydrogen Production Activity of ZnCdS. *Chem. Eng. J.* **2021**, *425*, 131478. https://doi.org/10.1016/j.cej.2021.131478

2. Dung, V.D.; Thuy T.D. N.; Periyayya, U.; Yeong-Hoon, C.; Gyu-Cheol, K.; Jin-Kyu, Y.; Duy-Thanh T.; Thanh Duc, L.; Hyuk, C.; Hyun You, K.; Yeon-Tae Y.; In-Hwan, L. Insightful Understanding of Hot-carrier Generation and Transfer in Plasmonic Au@CeO_2 Core–Shell Photocatalysts for Light-Driven Hydrogen Evolution Improvement. *App. Catal. B. Envirom.* **2021**, *286*, 119947. https://doi.org/10.1016/j.apcatb.2021.119947

3. Rusinque, B.; Escobedo, S.; de Lasa, H. Hydrogen Production via Pd-TiO_2 Photocatalytic Water Splitting under Near-UV and Visible Light: Analysis of the Reaction Mechanism. *Catalysts* **2021**, *11*, 405. https://doi.org/10.3390/catal11030405

4. Chengzhang, Z.; Yuting, W.; Zhifeng, J.; Fanchao, X.; Qiming, X.; Cheng, S.; Qing, T.; Weixin, Z.; Xiaoguang, D.; Shaobin, W. CeO_2 Nanocrystal-modified Layered MoS_2/g-C_3N_4 as 0D/2D Ternary Composite for Visible-Light Photocatalytic Hydrogen Evolution: Interfacial Consecutive Multi-Step Electron Transfer and Enhanced H_2O Reactant Adsorption. *Appl. Catal. B. Envirom.* **2019**, *259*, 118072. https://doi.org/10.1016/j.apcatb.2019.118072

5. Donghyung, K.; Kijung, Y. Boron Doping Induced Charge Transfer Switching of a C_3N_4/ZnO Photocatalyst from Z-Scheme to Type II to Enhance Photocatalytic Hydrogen Production. *Appl. Catal. B. Envirom.* **2021**, *282*, 119538. https://doi.org/10.1016/j.apcatb.2020.119538

6. Bashiri, R.; Irfan, M.S.; Mohamed, N.M.; Sufian, S.; Ling, L.Y.; Suhaimi, N.A.; Samsudin, M.F.R. Hierarchically $SrTiO_3$@TiO_2@Fe_2O_3 Nanorod Heterostructures for Enhanced

Photoelectrochemical Water Splitting. *Int. J Hydrogen Energy* **2021**, *46*, 24607-24619. https://doi.org/10.1016/j.ijhydene.2020.02.106

7. Tian, Y.; Yang, X.; Li, L.; Zhu, Y.; Wu, Q.; Li, Y.; Ma, F.; Yu, Y. A Direct Dual Z-scheme 3DOM SnS_2–ZnS/ZrO_2 Composite with Excellent Photocatalytic Degradation and Hydrogen Production Performance. *Chemosphere* **2021**, *279*, 130882.

8. Ma, B.; Bi, J.; Lv, J.; Kong, C.; Yan, P.; Zhao, X.; Zhang, X.; Yang, T.; Yang, Z. Inter-Embedded Au-Cu_2O Heterostructure for the Enhanced Hydrogen Production from Water Splitting under the Visible Light. *Chem. Eng. J.* **2021**, *41*, 126709. https://doi.org/10.1016/j.cej.2020.126709
